# Health impacts of extreme weather events – Cascading risks in a changing climate

**DOI:** 10.25646/11652

**Published:** 2023-09-06

**Authors:** Carsten Butsch, Liza-Marie Beckers, Enno Nilson, Marieke Frassl, Nicole Brennholt, René Kwiatkowski, Mareike Söder

**Affiliations:** 1 University of Bonn, Germany Department of Geography; 2 University of Cologne, Germany Institute of Geography; 3 Federal Institute of Hydrology Koblenz, Germany; 4 North Rhine-Westphalia State Office for Nature, Environment and Consumer Protection Department of Water Management and Protection Düsseldorf, Germany; 5 Federal Office for Civil Protection and Disaster Assistance Department for Risk Management, International Affairs Bonn, Germany; 6 Johann Heinrich von Thünen Institute Coordination Unit Climate and Soil Braunschweig, Germany

**Keywords:** VULNERABILITY, FLOODS, STORMS, DROUGHTS, FIRES, CLIMATE CHANGE ADAPTATION

## Abstract

**Background:**

Extreme weather events represent one of the most tangible impacts of anthropogenic climate change. They have increased in number and severity and a further increase is expected. This is accompanied by direct and indirect negative consequences for human health.

**Methods:**

Flooding events, storms and droughts are analysed here for Germany from a systemic perspective on the basis of a comprehensive literature review. Cascading risks beyond the initial event are also taken into account in order to depict downstream consequences.

**Results:**

In addition to the immediate health burdens caused by extreme weather events such as injuries, long-term consequences such as stress-related mental disorders occur. These stresses particularly affect certain vulnerable groups, e.g. older persons, children, pregnant women or first responders.

**Conclusions:**

A look at the cascading risks described in the international literature allows us to develop precautionary measures for adaptation to the consequences of climate change. Many adaptation measures protect against different risks at the same time. In addition to planning measures, these include, above all, increasing the population’s ability to protect itself through knowledge and strengthening of social networks.

## 1. Introduction

Extreme weather events are among the most tangible impacts of climate change in public perception. Individual events that trigger disasters are often explained by climate change. This is difficult from a scientific perspective, because while climate change alters the probability of the occurrence of extremes, this may not be a sufficient explanation for the individual event in question (Info box). However, there are many indications that at least some types of extreme weather events have increased in frequency and magnitude due to anthropogenic climate change [1]. This is accompanied by negative consequences for human health – both directly and indirectly.

Extreme weather events can be defined in various ways, with two definitions being established in the field of climate impact research. In the first, rarity and high magnitude is crucial, e.g. a statistically expected reoccurrence after 100 years or more. For the second, the consequences of the event for human society (i.e. health) are important, where these events disrupt social, technical or environmental systems [2, 3]. Below, both points of view are linked.

First, the climate change-induced change in frequency of potentially health-threatening events such as floods, storms, droughts, and fires is presented. Heatwaves are excluded here, as they are the subject of a separate article in this status report by Winklmayr et al. [4]. Based on this, the consequences of these events are analysed along risk cascades and direct and indirect impacts are systematically presented.

Extreme weather events are defined here as a ‘dynamic occurrence within a limited timeframe that impedes the normal functioning of a system’ [2, P. 4]. They trigger disasters when they encounter vulnerable social conditions and damage people, infrastructure, the economy or the environment to such an extent that external assistance becomes necessary (based on [5]). The Intergovernmental Panel on Climate Change (IPCC) dealt with vulnerability and resilience to extreme weather events in a special report [5]. Human health is conceptualised there as a vulnerable good and as potentially increasing vulnerability, since people with pre-existing conditions are often more affected by extreme weather events.

**Info box Evidence: The increase in extreme weather events as a result of climate change**

The sixth report of the Intergovernmental Panel on Climate Change (IPCC) asserts in its central statements that anthropogenic climate change is already having an impact on many weather and climate extremes in all regions of the world and that the evidence for attribution to human influence has strengthened in recent years [8].

However, not every extreme weather or hydrological phenomenon can be attributed to climate change. According to the conventions of the World Meteorological Organization (WMO), this attribution is only possible when system variables (e.g. temperature, precipitation or flood parameters, here: extreme values) shift noticeably in the multi-year mean [9]. This proof is difficult to provide due to the high natural variability in the climate system, usually quite short observation series and the rarity of extreme weather events. Since the climate fluctuates on multi-decadal time scales even under natural conditions, it is particularly difficult to clearly detect the share of anthropogenically enhanced climate change. For Germany, a change can be detected in relation to the event types heat, drought, storm surge and river flood (past analysis, data mostly since 1950), although the robustness – i.e. the unambiguity with which climate change can be identified as the reason for the changes – of the detected changes decreases in the aforementioned order. Almost all common heat indicators show significant changes with ever new extreme values [4, 10], which can partly be attributed to the anthropogenic contribution to climate change [11]. Established trends for droughts are more or less pronounced depending on the drought indicator. While meteorological indicators such as the climatic water balance or the forest fire index show comparatively clear changes all over Germany [12, 13], significant trends in hydrological indicators only emerge regionally [14]. This is partly due to compensating effects within the hydrological system, e.g. through water management or glacial melt. With regard to river floods, increases in annual maximum discharges can be observed at many gauges. In the case of extreme floods with a 100-year return probability, corresponding evidence is often not available (e.g. [15]). A similar picture emerges for North Sea gauges with regard to storm surges: while annual storm surges are increasing in magnitude, no trend can be discerned for ‘very severe storm surges’ due to a lack of past events and data [16].

In general, the more extreme and thus rarer an event under consideration, the more the limited length of observation series influences the possibilities for detecting changes. Therefore, it is difficult to reliably prove changes in the occurrence of extreme and destructive heavy rain or flash flood events and storms [17]. The required spatiotemporally high-resolution data series are only available for recent decades. However, this does not mean that climate change does not cause changes in these variables. By applying climate models, it could be shown, for example, that precipitation events such as the one that triggered the flood disaster in western Germany and Belgium in July 2021 have become more likely due to anthropogenic climate change [18].

In view of the current state of knowledge, despite uncertainties, it can be assumed for precautionary reasons that meteorological and hydrological extreme weather events in Germany will continue to increase in magnitude and frequency as climate change progresses (Info box) [1, 6]. The evidence is more robust for temperature- and sea-level-driven impact chains than for precipitation- and wind-driven impact cascades and greater for spatiotemporally large-scale phenomena such as droughts than for small-scale phenomena such as heavy rain or tornadoes. The greater the anthropogenic contribution to climate change will be, the higher the magnitude of the expected increase. Extreme weather events, however, are possible under all projections, with varying degrees of probability. The introductory article to this status report explains the basics of the different climate projections [7].

The effects of these events on human health are described below, based on a comprehensive evaluation of scientific literature, which was searched through Web of Knowledge, PubMed and Scopus. In particular, systematic reviews and meta-analyses were included. The text does not reproduce all sources found, but represents a focussed selection, which cannot meet the requirements of a systematic review.

## 2. Health impacts of selected extreme weather events

In this section, first the theoretical perspective on cascading risks is outlined. It is then applied to the extreme weather events under consideration – floods, storms, droughts and fires – in order to systematically illustrate the health impacts of these events. Finally, the extent to which vulnerable groups are particularly affected by the consequences of different extreme weather events is considered.

### 2.1 Cascading risks – conceptual foundations

The International Decade for Natural Disaster Reduction (1990–1999) led to intensive conceptual and theoretical works on risks. The United Nations Office for Disaster Risk Reduction was established as the leading institution; the Hyogo Framework and the Sendai Framework were adopted as internationally binding policy documents for risk reduction within the United Nations [19]. In parallel, new theoretical approaches emerged in the scientific discourse. Disasters are conceptualised as complex events in which the exposure of groups and systems and their vulnerability are analysed [19]. Accordingly, they are not the result of individual events, but arise from the interaction of different processes and circumstances [20]. Compound risks, which can trigger disasters that go beyond the impact of individual events, arise when (1) several extreme events occur simultaneously, (2) they encounter amplifying factors or (3) they are triggered by the unfavourable combination of several individually non-critical occurrences [20]. A special form are natural events that trigger technological failures and, as a consequence, disasters (NaTech events), e.g. the reactor disaster in Fukushima triggered by a tsunami.

Most recently, concepts of cascading risks have emerged that address the indirect effects of disasters. Through the interconnectedness of systems at local, regional and global scales, disturbances propagate and can be amplified, creating entirely new risks [20, 21]. This concept is based on the assumption of Complex Adaptive Systems (CAS). Complexity means that processes do not necessarily run in a linear fashion. Thus, unpredictable dynamics arise, because the number of connections between subsystems is very large and interactions are difficult to predict. As a result, small changes can have very large effects. When tipping points are crossed, CAS can reach new states of equilibrium. CAS are mostly dynamic and co-evolutions can occur when developments in individual subsystems influence developments in others [22].

The CAS perspective provides a framework for analysing the circumstances that lead to a disaster. Reducing vulnerability through adaptation measures can ideally prevent disasters or at least reduce their consequences, while active disaster management can prevent or at least limit the emergence of cascading risks.

This shows that to assess the health impact of extreme weather events, one must not only consider the immediate consequences of these events. A comprehensive analysis must also systematically examine the indirect and downstream consequences.

### 2.2 Deaths, injuries and monetary losses due to extreme weather events

Due to the complex interactions, it is not possible to fully assess the health impact of extreme weather events. Official statistics show causes of death according to the International Classification of Diseases (ICD); more detailed information is not collected. For example, if a person is killed by a falling tree, the cause of death statistics do not distinguish whether the tree fell due to a storm or due to another trigger. An alternative source of information is the Emergency Events Database (EM-DAT) of the Centre for Research on the Epidemiology of Disasters (CRED) [23]. Data from various sources on the health impact of worldwide disaster events since 1900 (including the extreme weather events considered here) have been collected and evaluated in this database. On the cut-off date of November 11, 2022, it contained 89 events for Germany, starting with a flooding event in the Danube region in 1920. A total of 63 storm events with 718 fatalities, 25 floods with 271 fatalities and one forest fire without any fatalities are documented. The database does not explicitly differentiate between storms and storm surges. The advantage of the EM-DAT database is the worldwide overview, but regional databases sometimes come to different results. The European Environment Agency recorded more than 4,700 deaths and damages amounting to 150 billion euros in 1,500 events between 1980 and 2013 [24]. Floods were the most frequent catastrophic events.

Table 1 shows the ten most serious events in Germany, based on the number of directly affected persons (fatalities and injured persons) registered in EM-DAT. Most injured persons were recorded for the heavy rain event that led to widespread flooding in mid-July 2021, mainly in Rhineland-Palatinate (RP) and North Rhine-Westphalia (NW), and the event claimed the second most lives in Germany with 197 fatalities. Most deaths occurred as a result of the storm surge of 1962 (347).

**Figure 1:**
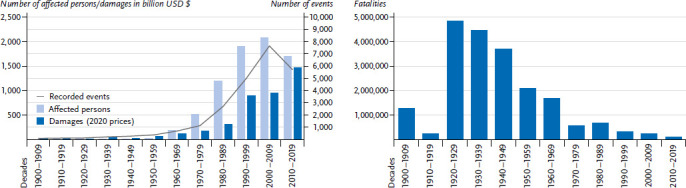
Global trends of documented loss events (event types considered in this review) since 1900 Source: Own representation based on EM-DAT [23] **Figure 1a (left)** Persons affected and monetary losses **Figure 1b (right)** Fatalities

In a global comparison, Germany’s exposure to natural hazards is relatively low and the risk profile differs, so that some globally relevant event types have not yet triggered any disasters here. Globally, 25,722 loss events with 38.4 million fatalities and 10.8 million injuries have been recorded in the EM-DAT database. The five events that have caused the most deaths globally in the last 122 years are (1) droughts, (2) epidemics and pandemics, with the COVID-19 pandemic not (yet) recorded in the database, (3) floods and inundations, (4) NaTech events and (5) earthquakes. At the same time, a decline in the number of fatalities can be observed from the 1930s onwards (Figure 1). Considering the growing world population and the increasing number of damaging events, this means a decreasing individual mortality risk, which is related to more effective risk management and improved international cooperation.

**Figure 2:**
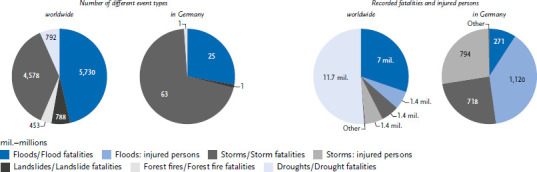
Persons affected by different types of events. Number of different event types worldwide and in Germany, recorded fatalities and injured persons from different types of events worldwide and in Germany. Source: Own representation based on EM-DAT [23]

For the extreme weather events considered below, the database shows 12,341 events worldwide with 20.2 million deaths (Figure 2). The largest single events are famines, which are triggered by floods or droughts. This represents a reduction in complexity that obscures interdependencies. The famine in Bengal in 1943, for example, is primarily recorded here as a drought, although Sen [25] showed that there was not a lack of food in Bengal, but that the poor population had no access to it. In Germany, storms and floods are the most common extreme weather events with the highest numbers of fatalities and persons affected (Figure 2).

With regard to extreme weather events, opposing trends can be observed globally: the number of events, persons affected and damage is increasing, while the number of fatalities is decreasing (Figure 1) [26]. For Germany, these trends are not equally clear. Due to the floods in July 2021, more deaths have already been recorded in the current decade than in the previous five decades. The highest insured losses were recorded in Germany for the decade 2000–2009.

### 2.3 Cascading risks due to floods, heavy rainfall and storm surges

Flood events can be triggered by various phenomena. Storm surges can occur in Germany when strong winds from northerly/north-westerly directions push water towards the coasts (North Sea and Baltic Sea) and this situation coincides with tidal flooding (mainly North Sea). River floods occur as a result of long-lasting and large-scale precipitation and possibly in conjunction with snow melting in the river catchment areas. Flash floods are the result of local heavy precipitation with high magnitudes, often within hours and in connection with a pronounced relief of the terrain (e.g. narrow valleys, large differences in altitude in a small area). Current knowledge suggests that all three event types (storm surges, river floods and flash floods) could increase in frequency and magnitude in the future (Info box, [6]). By the end of 2100, 3.7 million people could be affected by coastal flooding in Europe each year [27].

These events can cause great damage if they hit vulnerable groups or structures. Besides magnitude and duration of the events, local hydrodynamic conditions such as flow velocity in a cross-section or built-up areas in the channel determine the outcome [28]. The presence of risk management measures [27] and sources of hazard (e.g. industrial plants, landfills, sewage treatment plants, petrol stations [29, 30]) in the potential floodplains determines whether extreme events lead to damage.

**Figure 3:**
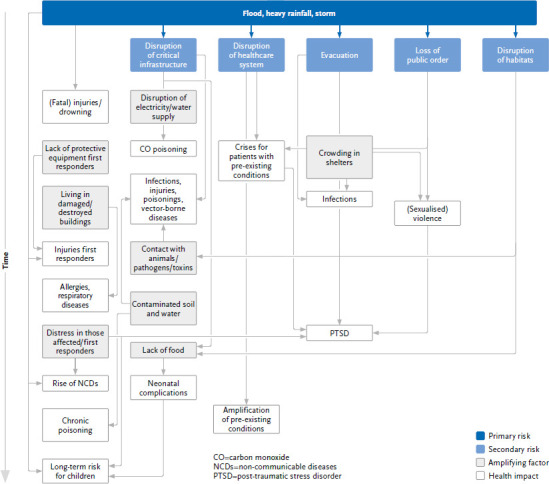
Cascading risks triggered by floods, heavy rainfall and storms. Arrows indicate possible causal relationships between risks, amplifying factors and health consequences. Source: Own representation

Immediate consequences for human health caused by the event may include deaths due to drowning, e.g. due to entrapment in buildings and vehicles, and (fatal) injuries. As a result of large-scale damage or flooding, there may be further deaths and other physical health consequences, e.g. from heart attacks, electrocution, fires, petrol and gas leaks (especially CO, CO_2_) due to technical defects and collapsing building components [28, 31, 32] (Figure 3, which also shows the cascading risks of extreme weather event ‘storms’, considered in Section 2.4 Cascading risks due to storms).

Indirectly, the disruption of critical infrastructures (including energy supply, water supply and disposal, transport and traffic, healthcare facilities) can lead to bottlenecks in medical care (such as through the cancellation of planned treatments, lack of medicines) and delays in disaster response and provision of essential goods (e.g. water, food, emergency shelters) [27, 31–33]. The relevant literature also describes an increase in cardiovascular complaints after flooding events [31, 34, 35]. However, it is not documented whether this is due to psychological distress during the event itself or to the failure of basic medical care. Other indirect health consequences due to increased exposure to heat, cold or damp rooms due to inadequate accommodation are not yet being systematically recorded. The development of mould in flood-damaged buildings can lead to respiratory diseases [30, 36]. Damage to drinking water and sewage infrastructure, as well as the failure of refrigerators due to power outages, can lead to an increased incidence of foodborne infections, the connection of which with climate change is considered in more detail by Dietrich et al. [37]. Vector-borne diseases may increase after floods when, for example, rodents seek shelter indoors [38, 39]. Another article in this status report is dedicated in detail to vector- and rodent-borne diseases as a result of climate change [40]. In addition, the loss of agricultural land due to flooding and erosion can threaten regional food production, and contamination from saltwater intrusion due to storm surges can affect drinking water supplies [41].

Heavy rainfall and flooding can lead to the discharge of pollutants and germs into water bodies via surface runoff, combined sewer overflows [42–44] and the destruction of wastewater infrastructure [41, 45]. In addition, pollutants, including persistent organic pollutants (POPs), heavy metals, pesticides, radionuclides and germs can be mobilised from sediments and polluted soils [29, 45]. Contact with contaminated water carries an increased risk of infections [29, 32], e.g. through the ingestion of antibiotic-resistant bacteria [46]. In Halle (Saale) in 2013, an increased number of infections with the parasite *Cryptosporidium hominis* was found in children who spent time in flood-plains and flooded meadows after a flooding event [47].

Medium-term health damage can be caused by exposure to pollutants via the air, e.g. in contaminated buildings, via water and via food intake. The latter are a consequence of the accumulation of heavy metals and POPs e.g. in arable soils and fish [28, 29]. However, directly observed effects after floods, such as headaches, dizziness, nausea, respiratory and skin irritation, could not yet be clearly attributed to a recorded increased exposure to halogenated pesticides (i.e. organic compounds in which at least one hydrogen atom has been replaced by chlorine, fluorine, bromine or iodine), volatile organic compounds, or heavy metals after flooding events [45]. When estimating the consequences, the limited data available is problematic, especially with regard to exposure before and after the event and the simultaneous recording of symptoms. In addition to the acute health consequences of event-related chemical exposure, it is particularly challenging to relate back to the potential chronic effects that only become noticeable several months after the event [45]. Many inorganic and organic pollutants are suspected of having carcinogenic, cardiovascular, neurotoxic, hepatotoxic, immunotoxic or reproductive effects [29, 48]. Due to the large number of pollutants, however, there are several research deficits.

A significant consequence of flooding events is the impairment of mental health [49, 50]. In Europe, increases in post-traumatic stress disorder (PTSD), anxiety disorders, depression and even suicides have been reported compared to the time before an event [32, 50]. These effects can be observed long after the event [50]. In addition to the direct traumatic experience of the event, the mental health consequences are also due to material losses and the often protracted reconstruction [51]. In a study on the consequences of the 2013 Elbe and Danube floods, the success of recovery correlated negatively with the length of time until the receipt of compensation payments, health status, financial status and obligations as property owner. Fear and anxiety due to (subjectively perceived) inadequate flood protection and the associated consequences of future events were also negative factors influencing recovery [51]. In the last 20 years, twelve flooding events in Germany have been registered in the EM-DAT database [23]. The floods in western Germany in July 2021 and the Elbe floods in 2002 and 2013 were particularly devastating.

On the German North Sea and Baltic Sea coasts, storm surges occur regularly, especially in the winter months. On the North Sea there have been 64 severe storm surges (>2.50 m above mean high water, mhw) since 1967, including 13 very severe storm surges (>3.50 m above mhw) [52]. However, effective coastal protection has been erected in many places. In particular, the experience of the storm surge in February 1962 (‘Hamburg storm surge’) led to increased coastal protection measures in Germany [53], so that the damage and health impacts of subsequent, more extreme events (e.g. 1976, 1990, 1994 and 2013 on the North Sea and 1995 and 2006 on the Baltic Sea) were greatly reduced [53].

### 2.4 Cascading risks due to storms

Large-scale storm events occur in Germany when large low-pressure vortices – cyclones – coming from the Atlantic pass over Central Europe. They can trigger winds of up to 200 km/h [54]. Among the most severe events observed in recent decades were cyclones Lothar (1999), Jeanett (2002), Kyrill (2007) and Zeynep (2022). In the EM-DAT database, a total of 63 storm events have been documented for Germany since 1900, 33 of which occurred since the year 2000 [23]. The greatest damage in the last two decades was caused by cyclone Kyrill in 2007. No clear trend in the development of storm events can be determined from past data. Although no reliable statements can be made, an increase in the frequency and magnitude of storm events must be expected in the future [54, 55]. In addition to large-scale storm events, approximately 20 to 60 tornadoes occur annually in Germany, which can cause severe damage on a small scale [56].

In a global comparison, Germany is less affected by severe storm events than countries in the tropics and sub-tropics, where tropical cyclones regularly trigger severe damage with high wind speeds and precipitation. This is also reflected in the literature on the health consequences of storms. A total of 22 review articles on health consequences of storms were identified, of which 14 were accessible and evaluated for this section. It becomes clear that there are large differences globally in terms of knowledge about the health consequences of extreme weather [34, 57]. The storm event whose consequences were analysed most thoroughly is Hurricane Katrina (2005, south-eastern USA).

The effects of storm on human health can be grouped into indirect and direct consequences at different levels (Figure 3). The direct health consequences of storms include injuries, for which comprehensive data are available in numerous studies [34, 58]. However, injuries also occur indirectly when first responders are injured during cleanup operations, during which they may also suffer poisoning [58, 59]. The stress to which storm victims are exposed during the event, but also the change in living conditions triggered by the event (e.g. homelessness, unemployment) manifest themselves in the medium term in an increase in non-communicable diseases (NCDs) [60]. The experience of stress also leads to long-term observable developmental delays in children whose mothers experienced a severe storm event during pregnancy. These delays also manifest as direct consequences through postnatal complications and are exacerbated – especially in the developmental context – by temporarily restricted access to food [61–63].

Indirectly, the failure of critical infrastructure causes negative health consequences, e.g. an increase in carbon monoxide poisoning when cooking indoors with wood, coal or gas during power outages [34, 58, 64]. Failures of water supply and sanitation can promote infections, and there is often an increase in unprotected contact with animals, whose faeces can carry pathogens, but which can also injure people through biting [34, 65]. As healthcare facilities are often inaccessible during storm events, critical situations can arise for people with pre-existing conditions, for example patients with chronic obstructive pulmonary disease (COPD), who are dependent on permanent oxygen supply, or patients requiring dialysis [60, 64]. Acutely insufficient healthcare for chronically ill patients can also manifest itself in a permanent deterioration of health [34, 60].

Severe storm events, such as tropical storms, force people to evacuate. Living together in crowded emergency shelters can encourage the spread of infectious diseases [62]. Flight, but also other traumatic events during the storm, can have long-term consequences for mental health, such as PTSD [62, 64, 66].

The loss of public order affects vulnerable groups in particular; in addition to children and older persons [67], women are often exposed to particular dangers (Section 2.6 Vulnerable groups and pathways of impact). There is evidence in the literature that women experience sexualised violence, which leads to further distress [61].

### 2.5 Cascading risks due to droughts and fires

For droughts, a distinction is made between three different types according to cause and consequence:

(1) Meteorological drought occurs when there is a combination of low precipitation and high temperatures. A high potential evaporation results in a negative climatic water balance (typical indicator).

(2) Agricultural drought describes the drought stress in agricultural crops due to a lack of water in the rooted soil. In north-western Europe, this only occurs after dry phases lasting several weeks. In extreme cases, this can lead to yield losses or even crop failures.

(3) Hydrological drought is recorded on the basis of water level data and is the result of a strained landscape water balance. Long and large-scale dry periods are the root cause for this type of drought as well.

Apart from the immediate effects of low water levels and water volumes, e.g. on drinking water availability, there are impacts on water quality and the risk of fires. Projections show that droughts in Central Europe could increase in frequency, magnitude and duration during the 21^st^ century [6]. Low precipitation, high temperatures and multiple demands could lead to increasing water stress, especially in summer and the transitional seasons.

**Figure 4:**
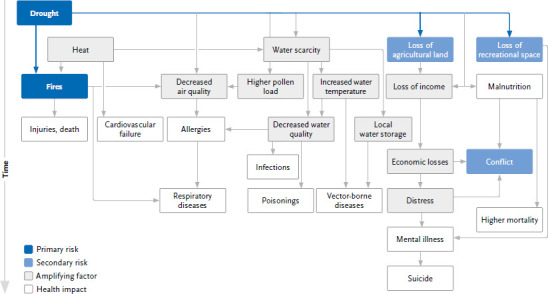
Cascading risks that can be triggered by droughts and fires. Arrows indicate possible causal relationships between risks, amplifying factors and health consequences. Source: Own representation

Cascading risks due to droughts can cause different health impacts (Figure 4). In extreme cases, they lead to malnutrition with increased mortality among vulnerable groups. This is mainly observed in the Global South [68]. In the Global North, a lack of food and drinking water supply currently poses little risk and the economic consequences dominate.

Droughts are usually accompanied by stable weather conditions and thus a reduced exchange of air masses. This leads to an accumulation of pollutants in the atmosphere and thus a deterioration of air quality with corresponding health consequences [69]. Elsewhere in this status report, the health impacts of climate change due to increased air pollutant loads are considered in more detail by BreitnerBusch et al. [70].

People working in agriculture are particularly exposed to the increasing agricultural droughts, often associated with heatwaves and strong sunlight, which is why potential health hazards such as heat stroke, cardiovascular failure and skin cancer especially affect this group of people [4, 71]. The economic uncertainties caused by droughts can also affect the mental health of people working in agriculture and forestry and increase the risk of suicide [72–75] (see also the scoping review by Gebhardt et al. [76] on the effects of climate change on mental health in this status report).

Low water levels can have a detrimental effect on water quality. Due to the reduced water volume and higher residence times of the water, it gets warmer and pollutants become less diluted [77]. High water temperatures and lower flow velocities during low water in summer are associated with the mass occurrence of potentially toxic phytoplankton (algal blooms), see also an article on waterborne infections and intoxications in this status report [78]. Direct contact with contaminated water occurs through occupational activities in and around water or recreational activities, e.g. water sports. For drinking water supplies in Germany, reduced water quality is a potential risk only in special cases. A possible increase in guideline value ex-ceedances may require more intensive drinking water treatment processes, e.g. in bank filtrates where enriched pollutants or toxins may not be sufficiently filtered out of the water. In drinking water reservoirs, toxin-producing cyanobacterial blooms can complicate water treatment. Contact with the contaminated water can lead to gastrointestinal infections and illnesses as well as zoonotic and vector-associated diseases [69, 78]. For contact with cyanotoxins, additional skin irritations and respiratory diseases have been reported, but are often not clearly attributable to cyanobacterial exposure [79].

Indirectly, droughts can lead to the spread of vector-associated diseases if, for example, in the absence of predators, mosquitoes multiply heavily in pools of water or in vessels for water storage [40, 80].

Droughts can also trigger health impacts as part of compound risks, e.g. when heavy rainfall events occur during a drought. On the one hand, infiltration of dry soils is inhibited, so there may be increased surface runoff and an increase in flash flood hazards and associated health impacts (Section 2.3 Cascading risks due to floods, heavy rainfall and storm surges). On the other hand, entry of pollutants or germs can lead to a deterioration of water quality.

Summer droughts are often accompanied by heatwaves. This provides ideal conditions for the occurrence of fires, which can be triggered by the slightest influences (e.g. by lightning or careless behaviour), thus increasing the risk of forest fires [81, 82]. In addition to climatic changes, other factors such as tree species composition (e.g. a high proportion of conifers) also play a role [6]. Forest and bush fires endanger the physical health of those affected as well as rescue workers directly through burns, through smoke development and the associated consequences for the respiratory tract, but also through effects on mental health or indirectly through disruption of infrastructure [83–85].

After 1959, which was an extremely dry year, the years 2003, 2018, 2019, 2022 had a particularly high precipitation deficit and drought periods, with additional regionally effective drought events [12, 14, 86]. The multi-year drought of 2018 to 2020 represents the most severe drought in Europe in the last 250 years [87].

No drought event is listed for Germany in the EM-DAT database [23] and in the recent past there have been no direct health effects of droughts in Germany documented in the literature. However, the hydrological droughts of recent years have led to pronounced low-water situations with observable deteriorations in water quality, e.g. due to massive phytoplankton blooms such as those observed recurrently in the Moselle since 2017 and in the Oder in 2022.

In the period between 1991 and 2021, the years 1991, 1992 and 2003 are those with the greatest number of forest fires [88]. The largest area was affected in 1992 (4,900 hectares), followed by 2019 (2,700 hectares) and 2018 (2,300 hectares) [88]. The European Forest Fire Information System (EFFIS) even assumes 3,600 hectares for 2018 and 4,300 hectares for 2022 [89]. In those regions in eastern Germany and in the Upper Rhine region, which are particularly affected by rising temperatures and droughts, more than 40 days with a high or very high forest fire risk are possible on average by the middle of the century [6].

### 2.6 Vulnerable groups and pathways of impact

The impact of extreme weather events differs regionally and for different population groups. Natural circumstances predispose a region for the occurrence of individual event types. Storm surges are a phenomenon of coasts and estuaries. Particularly strong winds can also occur there, as well as in exposed inland mountainous areas. Storm damage can also occur on a small scale where vulnerability is increased (e.g. forests, cities, vulnerable transport infrastructures such as overhead railway lines). River floods affect areas along waterways, flash floods can cause particular damage in areas with high relief. Extreme heavy rainfall events, however, can affect any place in Germany.

An increased risk of droughts and their potential consequences cannot be directly located, but there are different levels of impact depending on the type of drought (agricultural, hydrological). In regions and seasons with an already strained water balance, the consequences are more pronounced (e.g. eastern Germany) than in regions with some reserves in the system (e.g. Rhineland).

Four population groups are particularly affected by the health consequences of extreme weather events for different reasons:

(1) children, older people and people with physical limitations – they may not be able to care for themselves or get to safety and the physical stresses that occur may push them to their limits;

(2) people with low socioeconomic status – they are often directly exposed to extreme weather events and may have lower coping capacity;

(3) men are more often affected by the immediate consequences (e.g. higher risk tolerance);

(4) specific long-term consequences can occur for women (e.g. pregnancy complications).

The numbers of victims of the floods in western Germany and Belgium in July 2021 illustrate these overlapping vulnerabilities: among the immediate fatalities (184) in RP and NW, 138 persons (75%) were older than 60 years (population share in NW: 27%) and 3 (1.6%) were children under 14 years (population share in NW: 13%) [32, 90]. The ratio of men (65) to women (70) among fatalities was balanced in RP, while in NW about twice as many men (31) as women (18) died [32]. This is consistent with sources suggesting that men are less likely to take protective measures, such as evacuations [91]. The gender ratio in RP is consistent with patterns in storm surges (1953, 1962). The ratio in NW corresponds to the pattern of flood victims in Europe, the United States, and Australia [32]. People with physical or mental disabilities were particularly affected: twelve residents of a care facility died in their flooded living quarters [32].

Another vulnerable group are first responders. They are exposed to great physical dangers – through injuries, poisoning and great psychological strain. Disaster preparedness and post-disaster care can reduce the vulnerability of this group. An American study on the health risks associated with clean-up work after extreme events found that occupational fatalities occurred a median of 36.5 days after a storm (surge) event and were most common in clean-up (44%), restorative construction (26%), public utility restoration (8%) and preservation of law and order (6%) [92]. Animal bites are also described among rescue workers and animal owners [65].

## 3. Adaptation measures to increase resilience to extreme weather events

In order to increase resilience to extreme weather events, preventive precautionary and adaptation measures can be taken. These include measures that address different types of events as well as event-specific measures.

Self-protection is an important element of security provision for society as a whole. Since rescue forces cannot be everywhere at once during large-scale catastrophic events and may also be affected themselves, it may take some time until state assistance arrives. A population that has prepared for emergency situations in advance makes a significant contribution to coping with emergency situations collectively [93–95]. Social networks are an important asset for the emergence of spontaneous civil disaster relief, which is often of high importance in the first hours after a disaster [96]. Strengthening social networks in associations, faith-based institutions and through various forms of voluntary work is an abstract and difficult goal to achieve, but nevertheless an important building block of social resilience. As a first step, politics and society must recognise the importance of such networks for societal resilience in order to promote these institutions.

The Federal Office of Civil Protection and Disaster Assistance (Bundesamt für Bevölkerungsschutz und Katastrophenhilfe, BBK) offers various pages with recommendations for action responding to different types of hazards (e.g. heavy rain, storms, heatwaves) on its website [94, 97]. Likewise, timely risk communication and warning of the population is essential to minimise health impacts of extreme weather events. For this purpose, a mix of warning systems (e.g. sirens, mobile phone apps, cell broadcast – messages to all mobile phone users of selected radio cells) is used in Germany and is being further developed. The earlier the population is warned, the sooner they can prepare for the event and take precautionary measures or evacuate from an affected area [95, 98, 99].

Securing the water supply during and after extreme weather events is particularly important. Three areas of responsibility can be named for this: Water supply companies draw up action plans to maintain the supply. If the supply can no longer be maintained in the event of an incident, municipalities, through local disaster relief authorities among others, can (with the support of the district or the federal state) help with replacement supply measures (e.g. temporary laying of connecting pipes). If the extent of a supply failure increases, the Federal Government can contribute to the replacement supply (e.g. through self-sufficient wells, transport containers, mobile treatment plants) in accordance with §12 of the German Civil Protection and Disaster Relief Act. After flooding or during drought events, municipalities can issue orders to boil all drinking water to kill germs and thus ensure a safe drinking water quality [100].

In order to describe flood risks and damage potentials in Germany and Europe and to focus related measures, the EU directive on the assessment and management of flood risks [101] came into force on November 26, 2007 and was transposed into national law on March 01, 2010. Furthermore, a national spatial development plan for flood protection came into force on September 01, 2021 [102]. Likewise, heavy rain hazard maps can help to raise awareness among the population or help those responsible to take necessary structural measures [103].

In order to protect the particularly vulnerable group of emergency personnel in forest fires and to prepare them for operations, the German fire brigade association has published a recommendation on safety and tactics during vegetation fires [104]. The population can be informed about forest fire hazards and correct behaviour by means of information boards, flyers [81, 105] and other services such as the forest fire hazard index [106] and grassland fire index [107] of the German Meteorological Service (both currently available from March to October each year). In addition, silvicultural measures can be implemented to prevent forest fires, such as creating firebreaks or increasing the proportion of hardwoods in coniferous forests and reforesting with deciduous trees instead of conifers [105]. For early detection and suppression of forest fires, an automated early wildfire detection system is used in Lower Saxony, Brandenburg, Berlin, Mecklenburg-Western Pomerania, Saxony and Saxony-Anhalt [105, 108].

A political framework for strengthening Germany’s resilience to extreme weather events is provided by strategies that can be used to define and implement measures, e.g. the German Strategy for Adaptation to Climate Change, the German Strategy for Strengthening Resilience to Disasters and the National Water Strategy for Germany [109–111].

## 4. Discussion and conclusion

Extreme weather events, which already posed substantial health risks for Germany in the past, are expected to occur more frequently in the future due to climate change. The evidence is clearest for heatwaves, but hydrological events (heavy rain, floods, droughts) are also likely to increase. For storms, however, the evidence is less clear.

A key message at this point is that extreme weather events can only trigger disasters if they hit a vulnerable population and/or a vulnerable infrastructure. Although the complexity of human-environment systems makes it impossible to predict all interactions, adaptation measures can significantly reduce the risk. Many adaptation measures protect against different risks at the same time. In addition to planning measures, these include increasing the population’s ability to protect itself through knowledge and strengthening of social networks.

The healthcare system must be able to respond to extreme weather events on different time scales. In disaster situations, injuries and poisonings must be treated on site and it is necessary to ensure continuous care for those pregnant or with pre-existing conditions in order to minimise long-term consequences. When organising relief efforts, it is important to consider vulnerable groups and their needs. For this purpose, it would be important, for example, to know the residence of those people who cannot independently evacuate in the event of a disaster. In the medium and long term, the restoration of mental health is important, and healthcare resources must be earmarked for this. This also means that capacity building to respond to the challenges outlined here in the short, medium and long term must be part of climate change adaptation. In addition to disaster management, this also applies to the healthcare system, where necessary backup capacities must be created and permanently maintained.

One difficulty in recording the health impacts of extreme weather events is often inadequate data – both with regard to the events themselves and the health consequences. Especially the indirect consequences that unfold via cascading risks are not systematically recorded. For improved risk management, the creation of a database with comparable case studies would be an important knowledge base. This should integrate the different types of data and knowledge mentioned – from meteorological observations to descriptions of the event by the population – and thus enable the measuring of cascading effects.

In view of the available knowledge on future developments, it is advisable for all actors to review existing levels of protection. Authorities, the healthcare system, civil society and citizens must be aware of the shift in risks and actively adapt within their scope of action. Particular attention must be paid to vulnerable groups who cannot help themselves. How society deals with changing risks will pose major challenges in the coming decades. This includes negotiating responsibilities for preparedness and loss management. One important key to promoting social resilience in this context is empowering people to protect themselves – individually and in social networks.

## Key statements

- Climate change is expected to increase the frequency and magnitude of extreme weather events.
- Extreme weather events can trigger disasters through complex interactions with amplifying factors.
- Extreme weather events have a variety of indirect consequences that can be conceptualised as cascading risks.
- Existing data are insufficient for the detailed analysis of cascading risks.
- In addition to government agencies, the population must also be empowered to contribute independently to crisis management in the event of a disaster (self-protection).
- Various components of the risk management strategies for extreme weather events must be reviewed and, if necessary, adapted to climate change.

## Figures and Tables

**Table 1:** Compilation of the ten most serious events in Germany (direct health consequences) sorted by the number of affected persons recorded Source: Own representation based on EM-DAT [23]

Year	Event	Region/Place	Registered deaths	Registered injured persons	Number of affected persons recorded	Damages^1^	Insured losses^1^	Comment
**2021**	Flooding	BW, BY, HE, NW, RP, SN, ST, TH	197	1,000	1,197	40.0 bn	9.7 bn	
**1962**	Storm	HH, North Sea	347	Not specified	347	5.4 bn	Not specified	Storm surge
**1984**	Storm	Munich	3	250	253	2.5 bn	1.3 bn	Hailstorm
**2006**	Storm	BW, BY, HE	10	200	210	Not specified	Not specified	
**2007**	Storm	BB, BE, BW, BY, HB, HE, HH, MV, NI, NW, RP, SH, SL, SN, ST, TH	11	130	141	7.2 bn	4.1 bn	Cyclone (Kyrill)
**2002**	Flooding	BB, BW, BY, NI, SN, ST, TH	27	108	135	17.5 bn	2.7 bn	
**2006**	Storm	BW	1	100	101	Not specified	Not specified	Hail
**1972**	Storm	NI, GDR	54	Not specified	54	2.7 bn	Not specified	Cyclone (Quimburga)
**2020**	Storm	Frankfurt, Kiel, Cologne, Paderborn, Saarbrücken	0	33	33	Not specified	Not specified	Cyclone (Sabine)
**2017**	Storm	Altötting, Freyung-Grafenau, Passau	3	24	27	0.2 bn	Not specified	Hail

^1^ in US dollars, 2020 prices

BB=Brandenburg, BE=Berlin, bn=billion, BW=Baden-Württemberg, BY=Bavaria, GDR=German Democratic Republic, HB=Bremen, HE=Hesse, HH=Hamburg, MV=Mecklenburg-Western Pomerania, NI=Lower Saxony, NW=North Rhine-Westphalia, RP=Rhineland-Palatinate, SH=Schleswig-Holstein, SL=Saarland, SN=Saxony, ST=Saxony-Anhalt, TH=Thuringia
